# Approaches to the Modification of Perfluorosulfonic Acid Membranes

**DOI:** 10.3390/membranes13080721

**Published:** 2023-08-07

**Authors:** Ekaterina Yu. Safronova, Anna A. Lysova, Daria Yu. Voropaeva, Andrey B. Yaroslavtsev

**Affiliations:** Kurnakov Institute of General and Inorganic Chemistry, Russian Academy of Sciences, Leninsky Avenue, 31, 119991 Moscow, Russia; ailyina@yandex.ru (A.A.L.); voropaeva@igic.ras.ru (D.Y.V.); yaroslav@igic.ras.ru (A.B.Y.)

**Keywords:** perfluorosulfonic acid membrane, nanocomposite PFSA membrane, ion-exchange membrane, Nafion, proton conductivity, microstructure, hybrid membrane, modification

## Abstract

Polymer ion-exchange membranes are featured in a variety of modern technologies including separation, concentration and purification of gases and liquids, chemical and electrochemical synthesis, and hydrogen power generation. In addition to transport properties, the strength, elasticity, and chemical stability of such materials are important characteristics for practical applications. Perfluorosulfonic acid (PFSA) membranes are characterized by an optimal combination of these properties. Today, one of the most well-known practical applications of PFSA membranes is the development of fuel cells. Some disadvantages of PFSA membranes, such as low conductivity at low humidity and high temperature limit their application. The approaches to optimization of properties are modification of commercial PFSA membranes and polymers by incorporation of different additive or pretreatment. This review summarizes the approaches to their modification, which will allow the creation of materials with a different set of functional properties, differing in ion transport (first of all proton conductivity) and selectivity, based on commercially available samples. These approaches include the use of different treatment techniques as well as the creation of hybrid materials containing dopant nanoparticles. Modification of the intrapore space of the membrane was shown to be a way of targeting the key functional properties of the membranes.

## 1. Introduction

One of the most widely known polymeric ion-exchange membranes is perfluorosulfonic acid (PFSA) polymer membranes due to their unique transport properties and stability. The method of its production was developed in the late 1960s by DuPont. The product has been registered under the brand name Nafion^®^ [1]. It was intended to act as an insulating baffle to prevent back diffusion of the products in the chloralkali process. The efficiency of this process is related to the high selectivity of the cation transfer along with the strength and chemical stability of the membrane.

**Figure 1:**
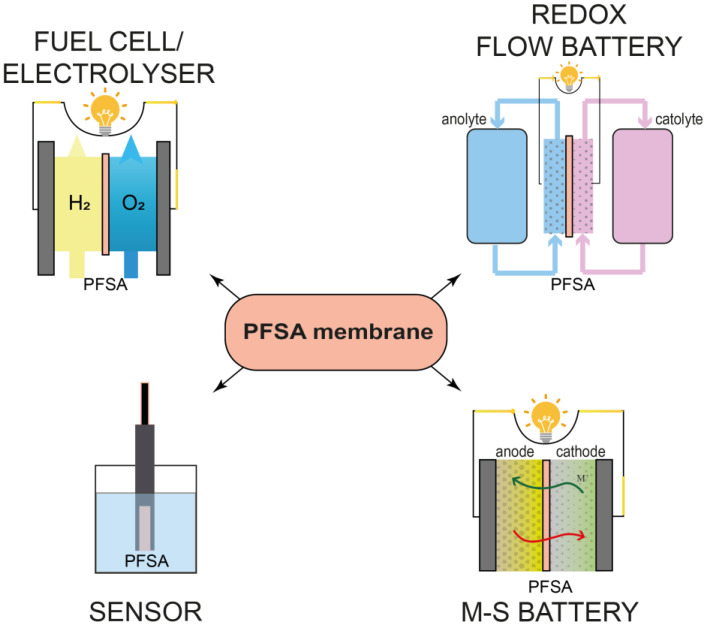
Examples of general directions of application of PFSA membranes.

Over the years, PFSA materials with different structures, ion-exchange capacities (IECs), thicknesses have been developed and are now commercially available, making them suitable for a variety of applications. PFSA membranes are widely used in energy generation and storage systems, particularly in fuel cells (FCs), electrolyzers, redox-flow batteries, metal-sulfur batteries, and sensors (Figure 1) [2,3,4,5,6,7,8,9]. PFSA membranes are most widely used in FCs, where they are used as a proton-conducting electrolyte and binder in catalytic inks [10,11,12,13,14,15,16,17].

The polymer membrane in FC performs the following functions: transfers protons from anode to cathode, inhibits electron transfer, and prevents the mixing of fuel with an oxidizer. Therefore, it should have the following properties: (i) high proton conductivity (especially at low humidity and high temperature), (ii) low gas permeability, (iii) low electron conductivity, (iv) high chemical stability (during FC operation, highly active peroxide radicals may be formed), (v) low rate of water transfer by diffusion and electroosmosis (to prevent flooding of the cathode of the FC and, consequently, power reduction), (vi) good strength in both dry and swollen state, and low volume swelling (to increase stability of membrane electrode assembly (MEA) during hydration/dehydration processes) [18,19]. From a commercialization point of view, the membrane should be relatively inexpensive and durable [20,21].

Another application of PFSA membranes is their use as an electrolyte in redox-flow batteries, the most common of which are vanadium redox-flow batteries (VRFB) [22,23]. For efficient VRFB operation, the membrane should provide rapid proton transfer, limit the transfer of vanadium ions, and have good chemical stability in acidic and oxidizing environments. To optimize the properties of PFSA membranes to improve their efficiency in VRFBs, they are modified by creating hybrid membranes in order to reduce vanadium ion crossover [24].

The use of PFSA membranes in metal-sulfur batteries as electrolyte can significantly improve the electrochemical characteristics of batteries during their long-term cycling. The membrane suppresses the diffusion of negatively charged polysulfides, which provides only a slight decrease in the battery capacity after long-term operation [9].

PFSA membranes are also used to create various sensors, such as potentiometric and amperometric sensors for the determination of oxygen, hydrogen, and water [25,26,27]. The biocompatibility of PFSA membranes makes it possible to produce sensors based on them for in vivo blood diagnostics, in particular, for determination of glucose, nitric oxide, hemoglobin, bilirubin, etc. [28,29,30,31]. Nafion membranes are also used to create optical sensors for moisture detection [32]. In this case, the approach is based on the equilibrium protonation-deprotonation reaction of the optically active substance. The wide range of applications of PFSA membranes in various modern technologies of high relevance maintains an active interest in them.

**Figure 2:**
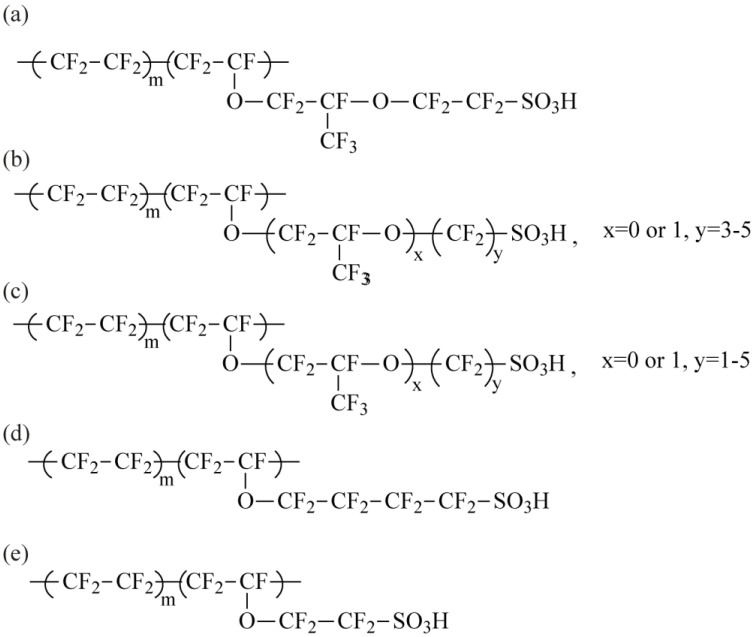
Structure of different PFSA membranes: (**a**) Nafion^®^ (du Pont de Numerous, Wilmington, DE, USA) and MF-4SC (PlastPolymer, Saint Petersburg, Russia), (**b**) Aciplex^®^ (Asahi Chemical, Tokyo, Japan), (**c**) Flemion^®^ (AGC, Tokyo, Japan), (**d**) 3M^®^ Polymer (3M Science, Two Harbors, MN, USA), and (**e**) Aquivion^®^ (Solvay, Brussels, Belgium) and Dow (Dow Inc., Midland, MI, USA).

The Nafion^®^ membrane is the most studied material among PFSAs [33,34,35,36,37,38,39,40,41] and now is considered a benchmark for comparing the properties of different polymer electrolytes for fuel cells (FC) and a number of other electrochemical devices. Nafion^®^ is a copolymer consisting of a partially crystalline polytetrafluoroethylene matrix and a side chain containing a terminal sulfonic acid group (Figure 2a). These membranes have high proton conductivity, as well as good chemical and thermal stability. Nafion^®^ is commercially available in different film thicknesses (Nafion^®^ 115, Nafion^®^ 117 produced by extrusion, and Nafion^®^ 212 produced by casting; Nafion^®^ N324 with improved mechanical properties arising from addition of polytetrafluoroethylene), dispersions, powder, and pellets [42], offering many opportunities to develop new materials based on it. MF-4SC (or MF-4SK in some references) is a Russian analogue of the Nafion^®^ membrane (Figure 2a).

**Figure 3:**
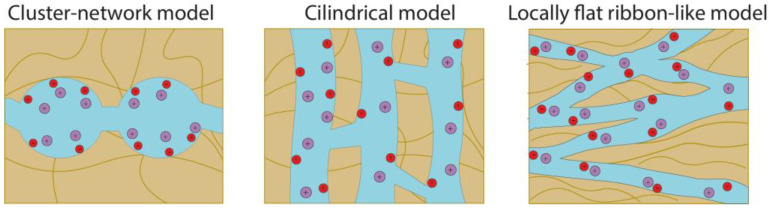
Schematic representation of the microstructure of PFSA membranes in the hydrated state according to different models [79,80,81]. Red circles mean negatively charged functional groups, violet circles mean positively charged counterions.

Other PFSA of various chemical compositions, with different equivalent weights (EW, g/mol, i.e., average polymer mass per functional group), and side chain lengths have become very popular in recent years. To date, PFSA membranes with an EW of 600 to 1500 are available [40,43,44]. The EW value determines the relationship between the transport properties and the stability of the material. As the EW increases, the strength of the PFSA membranes increases; however, in return, their conductivity decreases. Nafion membranes with an EW of 1100 are the most used membranes [40]. In the 1980s, Dow proposed a method for producing a polymer with a short side chain (Figure 2e) [45]. However, despite the good transport and mechanical properties of the resulting materials, multistep synthesis and expensive processing resulted in low interest in such membranes. Later, Solvay shortened the methodology for a similar polymer (Aquivion^®^) to 4 steps, which renewed interest for PFSA membranes with short side chain (Figure 2e) [46]. Currently, PFSA membranes are commercially available with different side chain lengths and EW, such as Aciplex^®^ (Figure 2b), Flemion^®^ (Figure 2c) and 3M^®^ (Figure 2d). Among the differences in PFSA membranes with a short side chain, a higher degree of crystallinity and a higher glass transition temperature compared to Nafion^®^ are often noted. The water absorption capacity of PFSA membranes with a short side chain and their high proton conductivity open wide prospects for the use of such materials in FCs [43,47,48,49,50,51,52]. The main features of microstructure as the well as sorption and transport properties of PFSA membranes of different chemical composition were summarized in the review by Kusoglu A. and Weber A. [40].

The most important advantages of PFSA membranes over other similar materials are chemical and thermal stability, as well as high ionic conductivity, high cation transport selectivity, and low gas permeability [3,40]. At the same time, the significant disadvantages of this type of materials include the dependence of conductivity on ambient humidity, as well as the limited operating temperature range of PFSA membranes, which significantly limits both their application in FC and the development of this field as a whole. Their modification or creation of hybrid materials by including dopants of different natures in the membrane matrix can be considered as an approach to directed change and optimization of the properties of the PFSA membrane [12,53]. To date, most of the research has been done with Nafion^®^. From a small number of commercially available membranes, modification makes it possible to be obtained with different conductive, sorption, and mechanical properties. This research began at the end of the twentieth century with the main aim of obtaining materials with desired properties. The requirements for new membranes were based on the shortcomings of commercial designs and initially mainly focused on the use in FC. Prominent trends in the optimization of PFSA membranes focused on a decrease in the dependence of their properties on humidity, increased proton conductivity at high temperature and low humidity, as well as an increase in operating temperature (above 100 °C). Modification of Nafion^®^-type membranes by the introduction of various dopants, mostly inorganic, was initially used as the main approach. Materials based on polymer and inorganic additives were called hybrid or composite materials. The results of this research have been summarized in a number of review articles [2,12,53,54,55,56]. Later, due to the accumulation of knowledge about the degradation mechanisms of materials and membrane-electrode assemblies (MEAs), much attention was paid to the stability of membranes (both unmodified and hybrid ones) and MEAs over time under conditions close to their operating conditions. To date, many studies have been published that focus on the effect of temperature, humidity, and applying mechanical stress on the properties of PFSA membranes.

The significant dependence of membranes properties on their prehistory (preparation and pretreatment conditions) is a very important feature of such PFSA membranes. These membranes are usually conditioned by various techniques prior to use to remove traces of impurities and convert them into proton form. Temperature, duration, and solution composition affect moisture content, ionic conductivity, diffusion permeability, as well as the mechanical properties and microstructure of PFSA membranes [34,57,58,59,60,61]. This results in a change in their properties, in particular proton conductivity and water uptake [62]. Only a few authors have considered this approach an opportunity to target change in membrane properties [63,64].

PFSA membranes can be modified by physical, physicochemical, or chemical processes: mechanical deformation, profiling, thermal and hydrothermal treatment, treatment with different solvents, introduction of dopants, and chemical surface modification, by changing the thickness of the film [65]. Many reviews describe advances in the field of hybrid materials, with a primary focus on different modification methods and materials for FC, among which PFSA membranes are highlighted in a single section [12,14,55,66,67,68,69,70,71]. In a recent review, the features of ion transport and water transfer in ion-exchange polymer membranes, including PFSA membranes, were discussed and approaches to modeling transport processes in such membranes, including hybrid membranes, were described [72]. At the same time, there are limited examples in the literature that discuss the possibility and reasons for changes in properties during modification of PFSA membranes as a result of physical and chemical treatment. The main distinction of this Review is the consideration of the possibilities of the influence of the properties of PFSA membranes by chemical and physicochemical action and the summary of the reasons for the changes in these properties from the viewpoint of the microstructure of PFSA membranes. The influence of modification without additives (treatment at different temperatures, humidity, mechanical loading, ultrasonic (US) treatment) and with incorporation of various additives will be described. Also, the influence of the nature of the dopant, concentration, and method of incorporation on the organization of the intrapore structure and membrane properties will be considered in the second case. Thus, this review outlines several approaches to direct modification of PFSA membranes by affecting their morphology using different processing methods and by creating hybrid materials containing dopant nanoparticles.

## 2. Microstructure of PFSA Membranes

The properties of PFSA membranes are largely determined by their microstructure. PFSA membranes are partially crystalline materials with a degree of crystallinity of ~20% [73]. The polymer is a partially crystalline copolymer of polytetrafluoroethylene, which forms the main chain, and a perfluorinated monomer containing sulfonated monomer, which forms side chains with terminated functional sulfogroups. The polytetrafluoroethylene main chain provides high stability and allows shaping at high degrees of swelling. The degree of crystallinity of PFSA membranes is lower than that of polytetrafluoroethylene and decreases with decreasing EW. At EW < 800 the polymer most often becomes completely amorphous. Side chains with terminated sulfogroups produce package defects, preventing crystallization of the polytetrafluoroethylene matrix.

There is a correlation between the microstructure of PFSA membranes and their swelling and transport properties [74,75]. The formation of internal microstructure due to self-organization processes caused by the presence of hydrophilic regions of polymer side chains and hydrophobic main chain is a distinctive feature of PFSA membranes. Several models of the PFSA membrane microstructure have been proposed in the literature, which vary depending on the chemical structure of the polymer (EW, side chain length) and external conditions (humidity, temperature). The specific features of the microstructure of PFSA membranes are described in detail in the review [40]. In this review, this point will be briefly outlined.

The hydrophilic region of the PFSA membranes is a system of pores connected by channels (Figure 3) [40,76]. The number of pores, their size, and degree of connectivity depend on the degree of water absorption of the membranes [77,78]. A cluster-channel model proposed by Gierke describes their structure based on the results of small-angle X-ray scattering studies of PFSA materials of different ionic forms and EW [77]. Although it is commonly recognized that hydrated Nafion is organized into distinct hydrophilic domains or clusters within the hydrophobic matrix, the geometry and length scale of these domains remain a subject of discussion. The cluster-channel model uses the average pore size, making this representation more convenient. In addition, other models have been proposed that describe the structure of PFSA membranes, the main ones being the cylindrical or layered model, the elongated polymeric aggregate model proposed by Loppinet, and the locally flat ribbon model discussed by Kreuer [40,79,80,81]. The locally flat ribbon-like model is considered to be one of the most favorable models for describing various properties of PFSA. This model considers elongated polymer aggregates with a locally flat interface (ribbon) placed in a continuous ionic medium. The morphology of Nafion demonstrates tortuosity on a scale of 30–100 nm, which is strongly influenced by pretreatment [79]. The main difference between the cluster-channel and alternative models is the shape and size of the clusters. A schematic comparison of the main models is shown in Figure 3.

**Figure 4:**
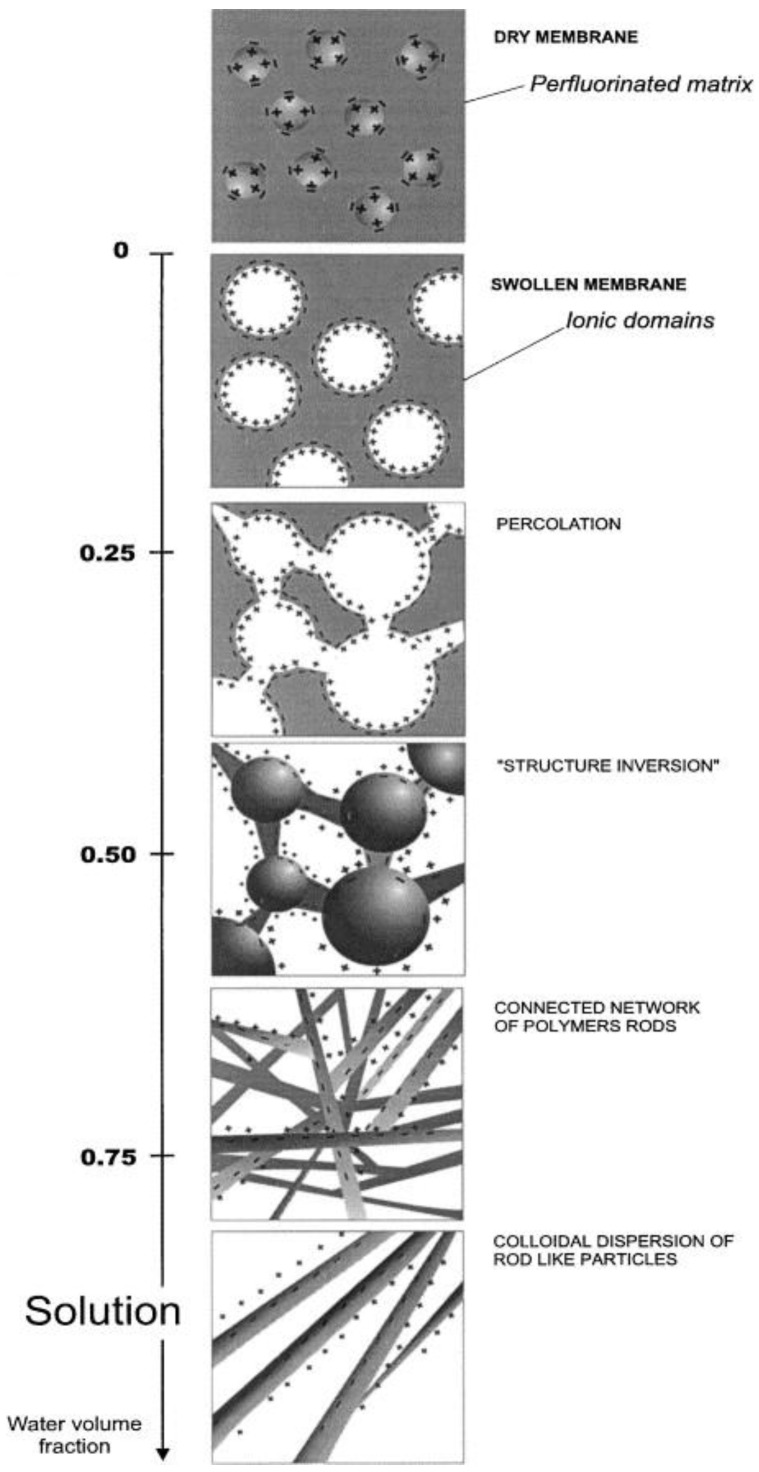
Schematic representation of the structural evolution depending on the water uptake. Reprinted from Ref. [78] with permission from Elsevier.

Gebel described the change in microstructure of PFSA membranes at different water uptake (Figure 4) [78]. In the dry state, only isolated pores are found in the membrane matrix. According to small-angle X-ray scattering data for Nafion^®^ 117 with EW = 1100, the diameter of the pores in the dry state is approximately 1.8 nm and each pore contains ~26 sulfogroups [76]. PFSA polymers are highly elastic polymers, so the change in pore size during membrane swelling is determined by the relationship between the internal osmotic pressure resulting from the repulsive forces during hydration and the Van der Waals forces of attraction and the elastic forces of the polymer matrix [78]. The increase in pore size during hydration is caused by their expansion and subsequent rearrangement. With increasing degree of hydration, the number of pores decreases, their size increases (their diameter reaches 5 nm for the membrane with EW 1100 in proton form and hydrated state) and the number of sulfogroups in them increases (up to 70 -SO_3_^−^ groups in one pore) [82].

## 3. Modification of PFSA Membranes by Different Treatments

This section presents a review of the works focused on the changes in the properties of PFSA membranes as a result of various types of modification without additives (treatment at different temperatures, humidity, mechanical loading, ultrasonic (US) treatment).

The prehistory of polymer materials affects their properties. For example, film formation and its pretreatment determine the packing of the polymer matrix and influence the permeability and selectivity of membranes, as well as their mechanical properties [83,84,85,86,87,88,89]. PFSA membranes are known to have a “memory” effect, which means that their microstructure, water uptake, and transport properties are determined by film formation and irreversibly change after treatment at different temperatures, humidity, and chemical composition of the reagents, as well as after mechanical loading [57,58,63,90,91,92,93,94,95]. The irreversible processes that occur during membrane treatment are associated with conformational transformations of the polymer and changes in their microstructure. At the same time, external action affects not only the main hydrophobic matrix, but also the hydrophilic regions, as well as the membrane surface [96]. The surface of PFSA membranes greatly determines their properties. Surface functionalization and profiling can be used to optimize their properties [10]. The principles outlined in this section are particularly important for membrane formation, pretreatment, and for assessing the effect of these parameters on membrane stability. The main results on changes in ionic conductivity of membranes due to different treatments are summarized in Table 1. As the conductivity values of the PFSA membranes change over a wide range [62], the properties of the treated membranes were compared with the initial samples.

Changing the surface shape of polymer films and its composition affects the functional properties [103,104]. Modification of the surface of PFSA membranes affects the rate of electromembrane processes by enhancing vortex fluxes near the surface [105] and at the three-phase interface (membrane-catalyst-gas diffusion layer) in the MEA, as well as improving proton transfer and water exchange. One of the most important problems in the formation of MEA of FC is to ensure proper contact between the membrane and the catalyst layer at the interface.

The surface profiling of the PFSA membrane is carried out by various methods, mainly by pressing or rolling with profiled plates, electron beam lithography with dry etching and ion-beam bombardment [10]. The use of electron beam assisted patterning and dry etching produces Nafion materials with highly accurate (<300 nm) line and circular surface patterns; however, this approach is expensive and difficult to implement [106]. When membranes are modified by pressing or rolling, die-shaped indentations of 500–1000 nm are formed on the surface. On the other hand, the advantages of this method are high reproducibility and ease of fabrication, allowing for scalable production of such materials. The use of Nafion^®^ 117 membranes with a surface modified by this method increases the proportion of active catalyst particles at the catalyst-electrolyte interface, proton transport, and MEA capacity [107]. In the case of membranes used in electrodialysis, profiling improves the performance of the process by enhancing the electroconvection of the solution near the membrane surface [108]. Ion-beam bombardment is a promising method because of its accessibility and lack of effect on the bulk of the membrane. This modification increases the surface roughness of the films, resulting in increased catalyst activity by increasing the FC interfacial area of the MEA and decreasing the resistance of the membrane [109]. This modification also reduces the permeability of the fuel (particularly methanol) by etching the sulfonic acid groups from the surface and increasing its hydrophobicity, as well as reducing the pore size [110]. However, a notable disadvantage of this method is the difficulty in controlling the shape of the profiled areas.

Thermal treatment of PFSA membranes increases their degree of crystallinity by ordering the main chain, creating a more homogeneous distribution of the crystalline phase, and by reducing the size of the ionic clusters [59,60,97,111]. Moreover, the rearrangement of ionic clusters can be caused by the weakening of electrostatic interactions and increased mobility of the side chains when treated at temperatures near or above the thermal transition temperature corresponding to the disorder of ionic clusters. Thermal treatment of PFSA membranes leads to an increase in their strength and affects their ability to absorb water [112]. Increased crystallinity of the hydrophobic matrix prevents an increase in membranes pore size during hydration; therefore, the higher the processing temperature, the lower the water uptake (degree of hydration per functional group λ(H_2_O/-SO_3_H) is 35 and 25 for Nafion^®^ membranes with EW = 1100 in proton form, obtained by casting at 130 and 190 °C, respectively [97]). For example, a 10% reduction in methanol permeability was reported through thermally treated Nafion^®^ membranes [113].

Thermal treatment of PFSA membranes in the hydrated state affects their properties differently depending on humidity, the physical state of the water in contact with them (gaseous or liquid) and temperature. During membrane treatment, two fast processes take place: (a) diffusion of water into/from and within the sample due to differences in osmotic pressure inside and outside; (b) a considerably slower process involving conformational transformations of the polymer [63,90]. Treatment of the membrane in contact with water vapor reduces water uptake and conductivity as such: the lower the relative humidity, the greater the difference between the properties of treated and the native membranes [63,92].

In contrast, hydrothermal treatment of PFSA membranes in the hydrated state in contact with liquid water leads to an increase in water uptake and ionic conductivity with increasing treatment temperature [63,90,92,100,114,115,116,117]. Treatment of PFSA membranes at temperatures near or above the glass transition temperature of the polymer leads to the softening of the membranes and to a reduction in Young’s modulus due to decrease of the crystalline phase proportion [118].

The US treatment of PFSA polymer dispersions is used for dissolution of polymer, preparation of hybrid membranes for homogenization of dopant nanoparticles in them, and preparation of catalytic inks for FC by employing polymer dispersion with catalyst nanoparticles. The following treatment leads to polymer deagglomeration and to a decrease in average molecular weight that is expressed as an irreversible decrease in dispersions viscosity with increasing duration and power of treatment [101,119,120]. Intense exposure to US leads to water sonolysis that generates hydrogen (H•) and hydroxyl (OH•) radicals [121]. Hydroxyl radicals attack the weakest tertiary carbons in the main and side chains, which can affect the polymer composition.

The impact of US treatment on the production of catalytic inks has been described in the literature; however, most of the research works are focused on the effect of such treatment on the catalyst [121,122]. Short-term treatment with US increases the electrochemical activity of the catalytic layer in FC, but longer exposure leads to dissolution of the metal nanoparticles and their separation from the carbon carrier [121].

Intensive stirring (≥10,000 rpm) of PFSA dispersions also reduces viscosity by more than 10% due to macromolecules deagglomeration [119]. At the same time, intense stirring of the polymer dispersion in the presence of catalyst nanoparticles can increase the electrochemical activity of the catalytic bed and improve the characteristics of the MEA [123].

The US pretreatment of PFSA dispersions allows for optimization of microstructure by increasing the mobility of macromolecule chains, the availability of functional sulfonic acid groups, and the connectivity of pores [101]; this increases conductivity by up to 40% and reduces the activation energy. However, it should be noted that this process decreases the selectivity of cation transfer, increases gas permeability, and produces carboxylic groups that can be easily attacked by free radicals generated during FC operation. To mitigate these processes, it is recommended to use low water-content aprotic solvents or dispersions [102].

Thus, the US treatment conditions of the PFSA dispersions is a good method for obtaining unmodified and hybrid membranes, as well as catalytic inks for MEA, and to determine the functional properties of the materials. The choice of thermal and US treatment conditions can improve and significantly deteriorate the stability of the materials, their sorption, and transport properties.

## 4. Hybrid PFSA Membranes

Modification of PFSA membranes by incorporation of additives into the membrane matrix offers great opportunities, since a small number of commercially available membranes can be used to obtain a huge number of materials with different properties by varying the nature of the dopant, the amount of added dopant, and the method of incorporation. For more than 30 years, many efforts have been directed towards the synthesis and study of hybrid organic-inorganic membrane materials [12,68,71,124,125]. Research interest in the field of hybrid membranes originally focused on electrolytes for FC, therefore many reviews and research articles on hybrid PFSA membranes focused on increasing FC performance by improving the sorption and transport properties of the electrolytes and expanding operational temperature and humidity ranges, as well as increasing their lifetime by increasing their chemical and mechanical stability [12,40,126]. The main results on the changes in the ionic conductivity of the membranes due to the creation of hybrid membranes are summarized in Table 2. As the conductivity values of the PFSA membranes change over a wide range [62], the properties of the hybrid membranes were compared with the initial samples.

To date, there are several techniques for producing hybrid membranes. The first and highly efficient, is the in-situ formation of inorganic particles in the membrane matrix [150,151]. For this purpose, the membrane is first treated with the precursor solution and then with an additional reagent (precipitant, oxidant, or reducing agent depending on how the dopant is produced). One or more treatment cycles can be performed to obtain hybrid membranes. The advantage of this method is the production of nanoparticles whose size is limited by the pore size of the membrane, which helps to increase the hybrid effect. The precursor penetrates the hydrophilic region of the membrane, and the addition of a second reagent leads to the formation of dopant particles in a limited space, as in the nanoreactor. However, the following method has many limitations; in particular, it allows the introduction of a limited set of dopants and does not allow one to achieve a high dopant concentration (usually no more than 3–5 wt.%). In some cases, an inhomogeneous distribution of the inorganic component is observed due to the presence of many larger particles on the membrane surface and in the near-surface layers compared to those located in the bulk volume [152]. In addition, the in-situ generation method is time-consuming as for bulk modification soaking in precursor and reagent solutions can take several hours to several days and requires a large number of reagents. In addition, some types of soluble compounds, such as heteropolyacids (HPAs), can be introduced into the membrane matrix by adsorption of the solution. The stability of the resulting material will depend on the strength of the interaction between the polymer and the inorganic component. The dopants introduced in this way eventually will be leached from the membrane by contact with liquid water; nevertheless, it is possible to stabilize them by various methods.

An alternative way to produce hybrid PFSA membranes is the casting of a polymer dispersion containing a calculated amount of precursor. The most technologically advanced modification method is casting in the presence of dopant nanoparticles. The advantage of this method is the possibility of obtaining modified membranes immediately after casting and independent direct control of the dopant composition. Any compound can be used as dopants, and the maximum number of injected particles is limited by the homogenization capability of the mixture and the mechanical properties of the materials. However, large particle size as well as a high degree of particles agglomeration can often reduce the efficiency of the modification. As noted above, intensive mechanical stirring or US treatment can be considered as a possible way of homogenizing the polymer and dopant solution. It is important to note that such pretreatment can affect the composition and properties of the formed materials.

Several research works described preparation of the PFSA hybrid membranes by other processes, in particular, the solid-phase process by hot-pressing films from a mixture of powder and dopant [153,154]. The following process is energy-consuming and inefficient for the production of membranes for FCs; however, it can be used to produce films of complex design and composition, in particular, gradient dopant thickness or area distribution, sandwich-type samples, films with profiled surfaces, etc.

Various compounds with different geometrical shapes (3D—spherical nanoparticles or 2D—nanotubes or fibers), degree of hydrophilicity and proton acceptor properties are used as dopants. Depending on the membrane modification method and the nature of the nanoparticles’ surface, the dopant may be located in the hydrophilic region, in the hydrophobic matrix, or partially in the hydrophilic region.

During the modification of the PFSA membrane by the in-situ method, the formation of hydrophilic dopant nanoparticles occurs in hydrophilic pores; however, in the case of casting, the pores are formed near the surface of the particles. As a result, the dopant occupies part of the volume inside the pores, while displacing the electroneutral solution localized in the center of the pores. The electroneutral solution contains a small number of counterions, but due to its remote location from the charged pore walls, it determines the transport of similarly charged coions and nonpolar molecules, such as gases [124,129]. Thus, the location of the dopant will affect the transport of anions and gas molecules.

**Figure 5:**
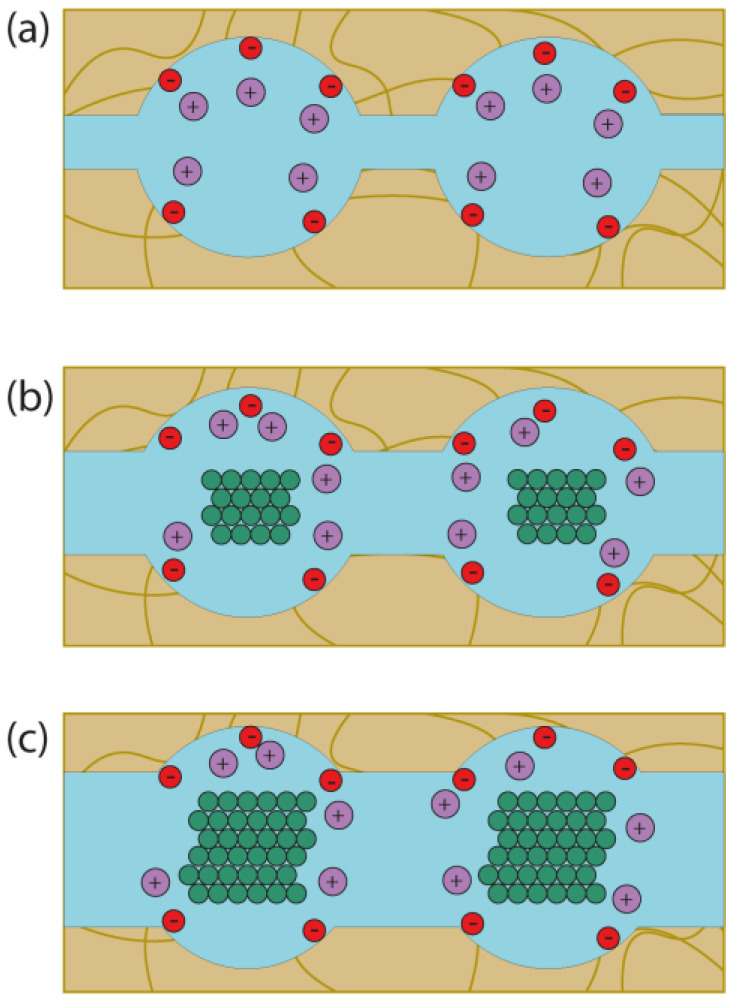
Schematic illustration of the model of limited pore wall elasticity of PFSA membranes. The scheme shows the structure of the pore and channel system of (**a**) unmodified membrane and hybrid membranes with (**b**) low and (**c**) high dopant content [155,156]. Red circles mean negatively charged functional groups, violet circles mean positively charged counterions, green means inorganic particles.

Based on a comparison of the conductivity and water uptake data, a model of the limited elasticity of membrane pore walls was proposed (Figure 5) [155]. According to this model, the introduction of nanoparticles into the membrane pores leads to their expansion. At the same time, the channels connecting the pores and limiting the membrane conductivity also expand [156].

The properties of hybrid membranes vary depending on the dopant nature. Hydrated metal oxides are used most frequently as dopants for PFSA membranes [12,125,127,128,129,131,132,133,134,157,158,159,160,161,162,163]. The advantages of such dopants are their high hydrophilicity and ability to retain water. Changing the nature of oxides affects their interaction with functional groups, and, hence, the properties of the membranes. Introduction of amphoteric oxides such as ZrO_2_ often results in a chemical interaction with sulfonic acid groups, leading to the exclusion of some functional groups from the ion exchange process, leading to a decrease in the water uptake and proton conductivity [164]. Moreover, the following process is accompanied by an increase in cation transfer selectivity. In contrast, the presence of nanoparticles with acidic properties will increase the concentration of charge carriers, the water uptake, and the proton conductivity of the membranes (including at low humidity) compared to the unmodified samples [164]. This leads to an increased capacity of MEAs based on hybrid membranes at low humidities. Therefore, most of the research works are focused on obtaining hybrid membranes containing hydrated silicon oxide that achieve high conductivity of membrane, due to high hygroscopicity of the dopant and chemical neutrality toward present functional groups. In some cases, the reduction of methanol crossover through such hybrid membranes is achieved [158]. The introduction of cerium oxide increases the chemical stability of hybrid membranes in the FC operating mode, as the dopant acts as a trap for hydroxyl radicals (•OH) [133,152,165].

The ability to functionalize the hydrated oxide surfaces to provide them with particular properties (change in the proton donor property or the degree of hydrophilicity) ensure the possibility to change the properties of hybrid membranes. The most widespread method is surface modification with acidic groups (most often -SO_3_H or -PO_3_H_2_) [136,137,152,166,167]. The increase in water uptake due to the introduction of proton-donating dopants leads to an increase in proton conductivity of the materials as compared to the materials containing oxides with an unmodified surface. The introduction of oxides with hydrophobic functionalized surfaces into the PFSA membrane matrix increases the proton conductivity and capacity of MEAs based on them by 25% as compared to the unmodified membrane despite the decrease of the water uptake [138,160,168].

Another group of dopants that can be used to modify PFSA membranes are proton-conducting particles, namely HPAs or insoluble acid salts. The hybrid effect is achieved by increasing the concentration of charge carriers and the self-proton conductivity of the introduced nanoparticles. The introduction of acidic zirconium and titanium phosphates into the PFSA membrane matrix increases proton conductivity and water uptake at low humidity and high temperature [140,169,170,171,172]. The possibility of increasing proton conductivity and operating temperature range has been reported in the literature. HPAs have the highest conductivity [173] among solid inorganic compounds; however, such compounds are highly soluble and, when introduced into the PFSA membrane matrix, they will be washed out during pretreatment and operation. The possible solution to this problem is to stabilize them on the surface of hydrated silicon oxide and/or convert them into insoluble salts [140,173].

Incorporation of dopants containing anions into the Nafion^®^ membrane matrix increases proton conductivity and reduces fuel crossover [141,174,175,176,177,178,179,180,181,182,183]. The possibility of increasing the conductivity and efficient operation of the membranes in the MEA at low humidity is very important [184]. The selectivity of proton transport (ratio of proton conductivity to permeability to methanol in direct-methanol FC) of hybrid membranes containing Cs_x_H_3−x_PW_12_O_40_ is six times higher than that of native Nafion, thus increasing the capacity of MEA [176]. When acidic dopants are introduced, a Debye layer is also formed near their surface, directed toward the layer formed by the pore walls. In addition to the protons from the dopant contributing toward an increase in conductivity, the additional Debye layer prevents the transfer of anions and neutral molecules such as hydrogen and alcohols through the membrane pores.

Hydrophobic additives, such as graphene oxide and carbon nanotubes (CNT), are also used as dopants [142,143,185,186]. Some studies have highlighted the possibility of increasing FC capacity by using hybrid membranes with such dopants [142,187]. However, in general, these dopants are added to PFSA membranes to improve mechanical properties and reduce the size difference between the swollen and dehydrated state of the sample [188,189,190]. The advantages of CNT-containing hybrid membranes include increased mechanical strength, Young’s modulus, and a significant decrease in methanol permeability; however, the proton conductivity is often decreased, and with high CNT content, the electronic conductivity value also increases, resulting in a loss of FC power. To improve conductivity, CNTs containing on their surface various proton-donating groups, such as carboxylic or sulfonic acid groups, can be used as dopants [145,190,191,192]. Increasing the concentration of charge carriers in the membrane matrix achieves increased proton conductivity and reduced fuel crossover, as well as improved FC performance [193]. Furthermore, if membranes are modified with hydrophobic CNTs, they are expected to be localized in the hydrophobic matrix, whereas CNTs which surface contains groups similar to the functional groups of the membrane can be expected to be at least partially localized in the hydrophilic region. The localization of the additive in the hybrid membrane matrix significantly affects their mechanical and transport properties and selectivity.

Metal nanoparticles are also used as dopants to produce hybrid PFSA membranes [146,194,195]. Introducing small amounts of metal nanoparticles may increase the proton conductivity of membranes despite the decreased water uptake [146]. The possibility of moisture retention in platinum-containing hybrid membranes in FC was reported in the literature [195]. The incorporation of polymers containing proton accepting groups (polyaniline (PANI), poly(3,4-ethylenedioxythiophene) (PEDOT)) into the PFSA membrane matrix affects their sorption and transport properties. Thus, modification of the PFSA membranes with PANI reduces the rate of undesirable transport (transport of anions and gases) [147,196]. Incorporation of PEDOT into the Nafion^®^ 117 matrix increases proton conductivity and decreases gas permeability compared to the unmodified membrane [147] which in return increases the capacity of MEA with the hybrid membrane by a factor of 1.5.

The introduction of the organic component (tetraethoxysilane) into the MF-4SC membrane, followed by a heat treatment, can suppress the electro-osmotic transfer of water [197,198]. The efficiency of concentration of electrolyte solutions by electrodialysis with ion-exchange membranes with high water uptake (λ(H_2_O/-SO_3_H) > 15) decreases due to the solvent transfer occurring not only as part of the hydrate shell of ions, but also due to transfer in the free form. Modification of PFSA membranes with tetraethoxysilane decreases the proportion of mesopores and leads to the prevalence of water transfer as part of the ions hydrate shell.

**Figure 6:**
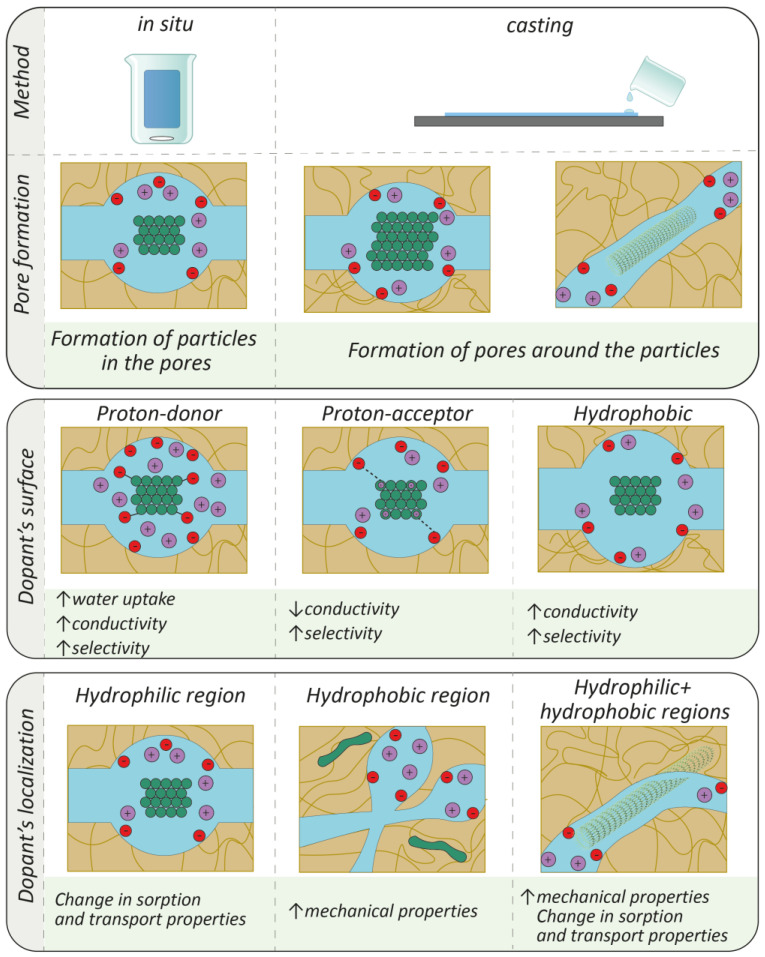
Schematic illustration of the ways for modification of membranes and general changes in the properties. Red circles mean negatively charged functional groups, violet circles mean positively charged counterions, green means inorganic particles.

Thus, the introduction of dopant nanoparticles into the PFSA membrane pores, which affect not only the pore and channel size, but also can contribute to the transfer process themselves through their own conductivity or interaction with functional groups, can achieve a significant change in the properties of the materials. Predicting the effect of modification on membrane morphology, in particular pore size and pore organization, is very important when selecting the dopant composition and the approach to obtaining hybrid materials that meet the requirements set forth. To this end, it is necessary to consider the following features of the modification (Table 3, Figure 6):

(i) method of obtaining a hybrid membrane;
(ii) prehistory of the PFSA (in the case of in situ modification, pretreatment, or membrane treatment; in the case of casting, the nature of the dispersing liquid and method of homogenization of the solution with the precursor or prepared dopant nanoparticles);
(iii) the amount of dopant;
(iv) dopant’s surface properties (acidity, hydrophilicity);
(v) morphology of the dopant.

## 5. Hybrid PFSA Membranes with Nonuniform Dopant Thickness Distribution

A large number of applications of ion-exchange membranes are determined by ion transport across their surface. Obtaining membranes with the surface layer modified with dopants of different nature opens up the prospect of creating high-performance materials with charge and specific selectivity, stability against radical oxidation, asymmetry of ionic transport, catalytic activity, etc. Two basic approaches are used to produce such materials: dopant synthesis in the surface layer of the PFSA membrane (gradient thickness distribution of particles without a distinct boundary of the modified layer) or deposition of a PFSA dispersion containing dopant particles directly on the film surface to form a modified layer and create a ‘sandwich’ structure.

Membranes with a gradient distribution of the dopant inside the sample are often characterized by asymmetric ionic transport. By doping the membrane with hydrated ZrO_2_, the difference in the diffusion permeability value in different directions reached 87%, and in some cases an increase and asymmetry of conductivity was observed [199]. At the same time, a barrier effect of the modified layer is observed, which appears to be associated with higher membrane permeability when the diffusion of acid/salt solutions occurs on the unmodified side.

A study of hybrid MF-4SC membranes with a gradient distribution of PANI shows an asymmetry in diffusion permeability and electrical conductivity determined at constant current from the voltametric curve [200,201]. The maximum value of the diffusion asymmetry reached 79% for dilute HCl solutions. The effect of the asymmetry of the voltametric characteristics depending on the orientation of the membranes to the ion flux was recently investigated [202]. A study of the voltametric curves in a series of isomolar HCl-NaCl solutions revealed that the PANI layer changes the mechanism of proton transfer when the modified side is oriented toward the counterion flux. An investigation of electro-osmotic and diffusion permeability, as well as membrane electrical conductivity in HCl solutions (with concentrations ranging from 0.01 to 2 M) demonstrated the blocking effect of the PANI layer which caused a considerable decrease in transport characteristics in a wide concentration range of solutions [203]. The use of MF-4SC/PANI hybrid membranes in the electrodialysis concentration of NaCl solutions provides a way to increase the salt content in the concentrate chamber by 50–70% compared to the basic MF-4SC membrane [204].

It was observed that during the synthesis of PANI directly in the PFSA matrix, PANI chains ‘grew’ through the membrane bulk and were localized not only on the surface (or surface layer), but also gradually distributed in the volume, penetrating to a certain depth [205]. The original method of MF-4SC/PANI production of PFSA membranes with controlled thickness of the modified layer was proposed [206]. The MF-4SC/PANI composites were found to have an improved electrochemical characteristic compared to samples with a gradient distribution.

The method of depositing a dopant containing PFSA dispersion on the membrane surface is more convenient in terms of controlling the thickness of the modified layer and the dopant content. Moreover, this method expands the range of possible dopants. Membranes doped by the ZrO_2_ layer-by-layer casting method exhibit a smaller effect of diffusion permeability asymmetry compared to the materials in which the dopant was introduced by in-situ method; however, there is a significant increase in their conductivity [199,207]. The possible reason for such a phenomenon is the influence of particles on the entire membrane microstructure when they are synthesized directly in the polymer matrix as well as increase in blocking effect in diffusion processes. In addition to that, differences in particle size may also have an effect. The maximum asymmetry effect for the membranes formed by layer casting was achieved at the 10 wt.% ZrO_2_ content in the modified layer and was 38% for hydrochloric acid solution [207].

Modification of the MF-4SC membrane by depositing modified PANI on the surface causes a diffusion permeability asymmetry that is more pronounced in dilute HCl solutions and reaches 53% for the 0.15 M molar PANI content in the modified layer [208].

The change in properties according to the thickness of the modified layer for hybrid Nafion membranes containing nanoparticles of rubidium or cesium acid salts of phosphorus-tungstate or silicon-tungstate HPAs in the modified layer was described in the literature [209]. The highest conductivity was observed for materials with a layer thickness of 35–50% of the total membrane thickness. As the layer thickness increases, an increase of diffusion permeability asymmetry is observed. The following shows a barrier effect on ionic transport. The effect in the potential increase of FC power was revealed through the use of asymmetric membranes oriented with the modified side toward the anode.

The formation of a thin modified layer containing sulfonated cerium oxide by spraying a solution on both surfaces of the Nafion improves both chemical stability (owing to the resistance of CeO_2_ to radical oxidation) and significantly increases the proton conductivity, which rises from 0.112 Ohm^−1^ cm^−1^ for the native Nafion to 0.199 Ohm^−1^∙cm^−1^ for modified membrane at 80 °C [210]. Patterned mesoporous TiO_2_ microplates embedded in Nafion^®^ membrane on the anode side surface improves performance of FC at high temperature/low relative humidity [211].

Application of a thin layer of PFSA on the surface of low-cost heterogeneous membranes can significantly improve their conductivity and increase the efficiency of the electrodialysis processes. A possible development of this approach is the introduction of dopants into the near-surface layer. The presence of hydrated silicon and zirconium oxides, as well as PANI in the MF-4SC membrane layer applied to the surface of the heterogeneous cation-exchange membrane MK-40 increases ionic conductivity and diffusion permeability [212,213]. The presence of the thin PFSA layer on the surface of a heterogeneous membrane increases its hydrophobicity and the intensity of mass transfer at the overlimiting current [214]. This can be explained by an increase in electroconvection. ‘Sliding’ water over a hydrophobic surface increases the tangential velocity of the electroconvective vortex [105,215]. Incorporation of various dopants, in particular CNTs, into the Nafion layer further enhances the efficiency of heterogeneous membranes in the electrodialysis process [216]. Introduction of cerium oxide increases membrane conductivity in various ionic forms and selective anion transfer [217]. Incorporation of titanium oxide (IV) into the applied surface layer increases the limiting current density by promoting electroconvection due to the change of the geometric heterogeneity of the membrane surface, increasing the mass transfer during electrodialysis [218,219].

The simultaneous introduction of silicon and titanium oxides, including those with modified surface, into the PFSA layer on the surface of heterogeneous membrane MK-40 leads to optimization of surface properties for the promotion of electroconvection (high surface charge while maintaining a sufficiently low degree of its hydrophilicity) which increases mass transfer and reduces membrane scaling during electrodialysis by inhibiting water splitting and inducing the hydrodynamic factor capable of flushing the deposits from the membrane surface [220].

Thus, the creation of hybrid PFSA membranes has an effect on their moisture content, proton conductivity, diffusion and gas permeability and mechanical properties. Varying the method of modification, nature, and amount of dopant allows one to obtain materials with different properties. At the same time, obtaining materials with desired properties requires a multifactor investigation of the effect of particle modification with different composition, size, and surface conditions on the properties of the obtained membranes.

## 6. Conclusions

PFSA membranes have a number of advantages that have stimulated interest in them for a number of decades. Their unique structure makes it possible to change their transport or mechanical properties through the use of various processing methods. In this case, the memory effect is realized, i.e., as a result of mechanical loading or thermal treatment of PFSA under different humidity conditions, their properties change significantly. The reason of the change in the properties of PFSA membranes is influence of the system of pores and channel: their interconnectivity, shape and size of pores and channels. If such an effect occurs as a result of a significant increase of the temperature and a decrease in humidity during operation, it is a negative factor, leading to accelerated degradation and deterioration of the properties. Furthermore, the impact of the prehistory of PFSA membranes, which can make a significant contribution to the change of properties, must be considered. Understanding the relationship between the prehistory of PFSA membranes, their properties, and the reason for their changes allows, on the one hand, directed modification by treatment of such materials to optimize their properties, and, on the other hand, makes it possible to predict changes of their properties during their operation in various processes.

Films of <50 µm thickness, which are required for high-performance FCs, can only be produced by casting from PFSA dispersions. The nature of the dispersing liquid or the method of dispersion pretreatment (ultrasonic treatment, stirring) has a crucial influence. Varying these parameters makes it possible to influence the morphology of the polymer and the properties of the resulting membranes, which improves their conductivity. The use of aprotic liquids as a solvent for the PFSA polymers provide the formation of membranes with good morphology due to the less aggregation of polymer. Moreover, the absence of water in the PFSA polymer dispersion provides less degradation of polymer upon sonication. Due to the previous sonication of the PFSA polymer solutions with the following membranes recast, proton conductivity increases by 40-45% as a result of the improved interconnectivity.

Hybrid membranes containing inorganic dopants offer a variety of opportunities to improve membrane properties. Varying their composition, size of dopant particles, and method of introduction make it possible to obtain materials with different properties in comparison to a small number of commercially available polymers. Modification predominantly affects the organization of the intrapore space (size and connectivity of pores and channels, distribution of ions in them), which in turn changes the water uptake, transport of ions, and nonpolar molecules. The most used dopants are hydrophilic hydrated oxides. Their incorporation provides increase in conductivity including at low humidification level. An additional increase in the number of charge carriers can be achieved by introducing inorganic acids, acid salts, or particles with functionalized surfaces containing proton-donor groups (heteropoly acids and their insoluble salts, oxides and CNTs with modifies surface). Incorporation of dopants with acidic properties leads to increase in water sorption, conductivity and decrease in the gas permeability. To date, many efforts have been made to identify the reasons for changes in material properties in terms of the microstructure features of hybrid membranes and the interactions between their components. This trend is relevant both the development of physicochemical and computational analysis methods and for the understanding of the properties of unmodified Nafion^®^ membranes. This review allows one to reveal the relationships between the composition, structure, and properties of hybrid membranes and to obtain materials with the desired properties. The solution of the above-mentioned problems contributes to the development in the field of obtaining new membrane materials, optimizing their properties and composition of components, increasing their lifetime, and simplifying the construction of devices based on them.

Thus, the targeted modification of PFSA polymers makes it possible to obtain materials with a wide range of different properties based on a small number of commercially available polymers. In terms of commercialization and use in real technological processes, it is necessary to address the issue of increasing the stability of PFSA-based materials, as well as, for several applications, the possibility of reducing the thickness of membranes. The most technological way to obtain hybrid and thin PFSA membranes is casting from polymer dispersions.

## Figures and Tables

**Table 1:** The main results on changes in ionic conductivity of membranes due to different treatments.

Processing Type	Processing Conditions	Initial Material	Proton Conductivity		Measurement Conditions	Distinctive Features	Ref.
			Modified Membrane	Initial Membrane			
Hot pressing	1335 N at a temperature of 135 °C for 20 min	Nafion 212	the in-plane conductivity: 160 mS/cm	the in-plane conductivity: 120 mS/cm	60 °C 100% RH *	Anisotropic conductivity: σ_in-plane_/σ_thru-plane_ ≈ 1.3	[91]
Chemical pre-treatment	heated in 0.5 M H_2_SO_4_ at 80 °C for 1 h	Nafion re-casted Nafion XL	22.76 mS/cm 55.44 mS/cm	4.89 mS/cm 50.55 mS/cm	25 °C	The pretreated membranes have higher water uptake, dimensional swelling ratios, and higher hydrophilicity, while the untreated membrane demonstrates a higher ion-exchange capacity.	[94]
	heated in DI * water at 80 °C for 1 h	Nafion Nafion XL	12.17 mS/cm 50.92 mS/cm	4.89 mS/cm 50.55 mS/cm			
	heated in 7 M HNO_3_ (HNO_3_:H_2_O = 1:1) at 80 °C for 30 min and in 1 M H_2_SO_4_ at 80 °C for 30 min	Nafion™ NR212	90 mS/cm	105 mS/cm	80 °C 100% RH	The untreated membrane has better stability of proton conductivity over time and the lower H_2_ crossover.	[96]
	heated in 5 vol.% H_2_O_2_ at 80 °C for 30 min and boiled in 1 M H_2_SO_4_ for 30 min	Nafion™ NR212	106 mS/cm	105 mS/cm			
Thermal treatment	heated at 30, 70 and 100 °C for 24 h in vacuum	Nafion 117	~190 mS/cm (30–70 °C) ~160 mS/cm (100 °C)	~190 mS/cm	80 °C in DI water	The drying temperature affects the reorganization within the ionic domains and proton conductivity. There is a significant decrease in the ionic aggregate size and a greater distance between aggregates with the increase in the treatment temperature.	[97]
	heated at 80 and 100 °C	MF-4SC	7.9 mS/cm (80 °C) 8.5 mS/cm (100 °C)	15.2 mS/cm	30 °C in DI water	Thermal treatment is accompanied by irreversible changes in the membrane matrix affecting the system of hydrophilic pores and channels.	[63]
	heated at 90 °C and RH 60/95%	MF-4SC-K^+^ form	3.5 mS/cm (RH 95%) 1.7 mS/cm (RH 60%)	5.3 mS/cm	30 °C in DI water		
	annealed at 120, 160, 200, and 240 °C under dry N_2_ for 1 h	Nafion with thickness 10, 30, 50 and 200 nm	5.9 mS/cm (200 nm, 240 °C) 2.7 mS/cm (50 nm, 240 °C) 1.1 mS/cm (10 nm, 240 °C)	23 mS/cm (200 nm) 7.9 mS/cm (50 nm) 2.3 mS/cm (10 nm)	25 °C 80% RH	Both the morphology and proton transport properties of the films were significantly changed after annealing of the films above the cluster transition temperature. For thin films, the smaller the thickness, the higher the temperature required to change the morphology.	[98]
	annealed for 20 h at temperatures from 80 to 220 °C	Aquivion-type polymer Nafion NR212 as reference sample	63 mS/cm (Aq 100) 100 mS/cm (Aq 120) 115 mS/cm (Aq 140) 135 mS/cm (Aq 170) 35 mS/cm (Aq 200)	120 mS/cm	50 °C 100% RH	Annealing of membranes at *T*_an_ = 170 ± 5 °C is optimal to achieve the best mechanical properties and proton conductivity	[99]
Hydrothermal treatment	Treatment in chamber with water at 140 °C	MF-4SC	18.5 mS/cm	15.2 mS/cm	30 °C in DI water	Treatment under hydrothermal conditions deteriorates the selectivity of cation transport	[63]
	Aging at 80 °C and RH 80%	Nafion 212	18 mS/cm after 300 days of aging	77 ± 1 mS/cm	Room temperature in DI water	The increase in the water content and RH accelerates the aging process. The decrease in the gas permeability coefficients observed for the aged membranes in comparison with that observed for the neat membranes.	[92,100]
US treatment	US treatment of the polymer solution before casting from 10 min to 24 h	Nafion solution	Maximum: 45 min US, 100% RH ~40 mS/cm; 30 min US, 32% RH ~7.5 mS/cm After 1440 min US ~31 mS/cm (100% RH) ~5 mS/cm (32% RH)	28 mS/cm (RH 100%) ~5.5 mS/cm (RH 32%)	30 °C in DI water and RH 32%	The dependence of conductivity of the membranes in the protonic form against the duration of sonication is not monotonic and pass the maximum at 30–45 min.	[101]
	low-frequency US treatment for 1 to 60 min and power from 2.7 to 9.4 W	Nafion	0.040 S/cm (10 min, 5.2 W) 0.024 S/cm (10 min, 9.4 W)	28 mS/cm	30 °C at RH 100% (in DI water)	The dependences of the proton conductivity of membranes on the power of ultrasonic treatment and its duration pass through a maximum at 5.2 W and 10 min.	[102]

* DI—deionized; RH—relative humidity.

**Table 2:** The main results on the changes in ionic conductivity of membranes due to the creation of hybrid membranes.

Dopant	Initial Material	Proton Conductivity		Measurement Conditions	Method of Particle Introduction	Ref.
		Modified Membrane	Initial Membrane			
SiO_2_	Nafion 212	33 mS/cm	24 mS/cm	110 °C RH 60%	The “swelling-filling” strategy of dopant introducing	[127]
	Nafion 115	63 mS/cm (MeOH); 65 mS/cm (EtOH) 60 mS/cm (i-PrOH)	58 mS/cm	80 °C RH 100 %	In-situ synthesis of nanoparticles in different sol-gel media	[128]
	Nafion 212	~32 mS/cm	~17 mS/cm	80 °C RH 60%	In-situ sol-gel synthesis	[129]
	Nafion	~140 mS/cm (water vapor) ~120 mS/cm (ethanol vapor)	~210 mS/cm (water vapor) ~80 mS/cm (EtOH vapor)	100 °C Under water and ethanol vapor	Casting of polymer dispersion with mesoporous silica	[130]
CeO_2_	Nafion	6 mS/cm (0.5 wt.% CeNT *) 4.8 mS/cm (1.0 wt.% CeNT) 5.1 mS/cm (1.5 wt.% CeNT)	4.1 mS/cm (recast Nafion) 4.0 mS/cm (Nafion NRE-212)	80 °C RH 20%	Casting of polymer dispersion with nanoporous CeO_2_ nanotubes	[131]
	MF-4SC	~25 mS/cm (1.3 wt.% CeO_2_) ~32 mS/cm (4.2 wt.% CeO_2_) ~42 mS/cm (5.5 wt.% CeO_2_)	~16 mS/cm	25 °C RH 32%	Casting of polymer dispersion with precursor for dopant synthesis	[132]
	Nafion	~18 mS/cm (1–5% CeO_2_)	8 mS/cm	60 °C RH 25%	Casting of polymer dispersion with the self-assembled Nafion–CeO_2_ nanoparticles	[133]
ZrO_2_	Nafion	~40 mS/cm (2.5 wt.% ZrO_2_) ~47 mS/cm (5 wt.% ZrO_2_) ~50 mS/cm (7.5 wt.% ZrO_2_)	~30 mS/cm (recast Nafion) ~44 mS/cm (Nafion 117)	100 °C RH 100%	Casting of polymer dispersion with ZrO_2_ nanoparticles	[134]
TiO_2_	Nafion	~37 mS/cm (16 vol.% dopant)	~100 mS/cm	100 °C RH 100%	In-situ formation of titanate nanotubes and nanorods	[135]
SiO_2_ with sulfonated surface	Nafion 212	17 mS/cm (RH 20%) 70 mS/cm (RH 60%)	12 mS/cm (RH 20%) 25 mS/cm (RH 60%)	110 °C RH 20–60%	In-situ sulfonation of Nafion/SiO_2_ membranes obtained with a “swelling-filling” strategy	[136]
TiO_2_ with sulfonated surface	Nafion	110 mS/cm (10 wt.% TiO_2_-S)	~40 mS/cm	140 °C RH 100%	Casting of polymer dispersion with sulfonated TiO_2_ nanoparticles	[137]
SiO_2_ with surface modified by groups with different nature	MF-4SC	36 mS/cm (3 wt.% SiO_2_/10 mol.% amino-groups) 25 mS/cm (3 wt.% SiO_2_/10 mol.%—(3-(2-imidazolin-1-yl)-propyl) groups) 90 mS/cm (3 wt.% SiO_2_/10 mol.% perfluorododecyl groups)	22 mS/cm	35 °C RH 100%	Casting of polymer dispersion with modified SiO_2_ nanoparticles	[138]
Acidic zirconium phosphate (ZrP)	Nafion 117	42 mS/cm (2.5 wt.% ZrP) 18 mS/cm (5 wt.% ZrP) 12 mS/cm (7.5 wt.% ZrP)	35 mS/cm	60 °C RH 70%	Impregnation method of hybrid membrane synthesis	[139]
Phosphotungstic acid/silica	Nafion 212	58 mS/cm	25 mS/cm	110 °C RH 60%	In-situ synthesis of PWA/SiO_2_ particles	[140]
M_x_H_3−x_PW_12_O_40_ and M_x_H_4−x_SiW_12_O_40_ (M = Rb and Cs)	Nafion	48.5 mS/cm (RbxH_3–x_PW_12_O_40_) 45.5 mS/cm (Cs_x_H_3−x_PW_12_O_40_) 61.6 mS/cm (Rb_x_H_4−x_SiW_12_O_40_) 68.9 mS/cm (Cs_x_H_4−x_SiW_12_O_40_)	39.5 mS/cm	40 °C RH 100%	Casting of polymer dispersion with 3 wt.% dopant	[141]
Graphene oxide	Nafion	17.3 mS/cm (0.5 wt.% dopant)	~5.5 mS/cm	80 °C RH 20%	Casting of polymer dispersion with dopant	[142]
Sulfonic acid functionalized CNT	Nafion	8 mS/cm (0.125 wt.% dopant) 23 mS/cm (0.25 wt.% dopant) 6 mS/cm (0.5 wt.% dopant)	2 mS/cm	60 °C RH 20%	Casting of polymer dispersion with dopant	[143]
Modified CNT	Nafion	~115 mS/cm (1 wt.% CNT and CNT@SiO_2_) ~123 mS/cm (1 wt.% CNT@SiO_2_-PWA) Reduced methanol permeability and enhanced selectivity for all hybrid membranes.	~123 mS/cm	60 °C	Casting of polymer dispersion with multiwalled CNT, CNTs, silicon oxide-covered carbon nano- tubes (CNT@SiO_2_) and phosphotungstic superacid-doped silicon oxide-covered carbon nanotubes (CNT@SiO_2_-PWA)	[144]
surface-sulfonated CNT	MF-4SC	5.5 mS/cm (1 wt.% CNT-SO_3_H) 4.4 mS/cm (1 wt.% CNT)	2.2 mS/cm	25 °C RH 32%	Casting of polymer dispersion with CNT and CNT-SO_3_H	[145]
Ag nanoparticles	MF-4SC	~40 mS/cm (10–12% Ag)	~13 mS/cm	80 °C RH 100%	In-situ synthesis of Ag nanoparticles	[146]
PEDOT	Nafion 117	~8.7 mS/cm	~8 mS/cm	85 °C RH 30%	In-situ chemical polymerization	[147]
PANI *	Nafion 112	10 mS/cm (polymerization in water) 20 mS/cm (polymerization in i-PrOH)	100 mS/cm	25 °C aqueous solution 2.5 M H_2_SO_4_	In-situ chemical polymerization in water or i-PrOH	[148]
PANI	Nafion 115	77.9 mS/cm (in-plane) 33 mS/cm (through-plane)	86.2 mS/cm (in-plane) 92 mS/cm (through-plane)	The in-plane ionic conductivity was measured in water, through-plane conductivity was measured in acid.	Multi-layered polyaniline/Nafion membranes	[149]

* CeNT—cerium oxide nanoparticles; PANI—polyaniline.

**Table 3:** Main features of hybrid PFSA membranes depend on the method of modification and the nature of the dopant.

Modification Method	
In-situ	Particle size of 3–7 nm; Dopant amount of 0.5–6 wt.% Limited range of dopants
Casting	Particle size of 3–15 nm (casting in the presence of precursor) and larger (casting in the presence of prepared nanoparticles) Possibility of increasing the dopant content Possibility of a broader choice of dopants
Dopant surface properties	
Enabling proton-donor capability	An increase in the number of charge carriers increases the moisture content, conductivity (especially at low humidity), and cation transport selectivity.
Enabling proton-acceptor capability	Binding of some of the sulfonic acid groups can lead to a decrease in proton conductivity and an increase in cation transport selectivity.
Increasing hydrophobicity	Possible localization in the hydrophilic region when obtained by casting in the presence of the precursor. Possible increase in conductivity and selectivity.
The shape of dopant particles	
Spherical	Particles are formed in the pores of the membranes (in situ) or the pores form around them (casting)
Extended (nanotubes or nanofibers)	It can be localized in both the hydrophilic and hydrophobic regions. Contribute to an increased strength
Localization of dopant	
Hydrophilic region	Changes in the sorption and transport properties of membranes.
Hydrophobic region	Weak impact on the sorption and transport properties of hybrid membranes. Possibility of improving mechanical properties.
Hydrophilic and hydrophobic regions simultaneously	Probable effects on tensile strength along with changes in the sorption and transport properties of membranes

## Data Availability

Not applicable.
